# A Survey on LPWAN Technologies in WBAN for Remote Health-Care Monitoring

**DOI:** 10.3390/s19235268

**Published:** 2019-11-29

**Authors:** Damilola D. Olatinwo, Adnan Abu-Mahfouz, Gerhard Hancke

**Affiliations:** 1 Department of Electrical, Electronic and Computer Engineering, University of Pretoria, Pretoria 0001, South Africa; damibaola@gmail.com (D.D.O.); ghancke@ieee.org (G.H.); 2 Council for Scientific and Industrial Research (CSIR), Pretoria 0184, South Africa; 3 Department of Computer Science, City University of Hong Kong, Hong Kong, China

**Keywords:** 5G, energy efficiency, health-care monitoring, LoRa, LPWAN, NB-IoT, Sigfox, WBAN, medical nanosensors, cloud computing

## Abstract

In ubiquitous health-care monitoring (HCM), wireless body area networks (WBANs) are envisioned as appealing solutions that may offer reliable methods for real-time monitoring of patients’ health conditions by employing the emerging communication technologies. This paper therefore focuses more on the state-of-the-art wireless communication systems that can be explored in the next-generation WBAN solutions for HCM. Also, this study addressed the critical issues confronted by the existing WBANs that are employed in HCM. Examples of such issues include wide-range health data communication constraint, health data delivery reliability concern, and energy efficiency, which are attributed to the limitations of the legacy short range, medium range, and the cellular technologies that are typically employed in WBAN systems. Since the WBAN sensor devices are usually configured with a finite battery power, they often get drained during prolonged operations. This phenomenon is technically exacerbated by the fact that the legacy communication systems, such as ZigBee, Bluetooth, 6LoWPAN, and so on, consume more energy during data communications. This unfortunate situation offers a scope for employing suitable communication systems identified in this study to improve the productivity of WBANs in HCM. For this to be achieved, the emerging communication systems such as the low-power wide-area networks (LPWANs) are investigated in this study based on their power transmission, data transmission rate, data reliability in the context of efficient data delivery, communication coverage, and latency, including their advantages, as well as disadvantages. As a consequence, the LPWAN solutions are presented for WBAN systems in remote HCM. Furthermore, this research work also points out future directions for the realization of the next-generation of WBANs, as well as how to improve the identified communication systems, to further enhance their productivity in WBAN solutions for HCM.

## 1. Introduction

In recent times, research efforts have been active in the field of wireless sensor networks (WSNs) to improve the health-care domain, and, to that effect, a new technology that is human-body-focused has been proposed. The new technology is referred to as wireless body area networks (WBANs). WBANs can therefore be described as a subfield of WSNs that is devoted to health-care monitoring (HCM). It is a new type of WSN communication paradigm that require its sensors to be placed in-body, on-body, or around the body [1]. WBAN technology has gained a lot of recognition because of its suitability and wide acceptance in both medical and nonmedical applications. Basically, some WBAN sensor devices, especially in medical applications, are dedicated to the continuous health monitoring of patients’ body without affecting their normal daily lifestyle [2,3]. It is noteworthy that WBAN solutions are sophisticated technologies that promise to advance HCM applications. Unfortunately, regardless of the interesting promises of WBAN solutions, they are faced with a number of issues considered as open research problems that need to be tackled to make WBANs efficient, effective, and reliable for HCM. Such issues include energy consumption, data communication reliability, and wide communication coverage. As a result of the mentioned issues, it may be difficult to meet some crucial quality of service (QoS) requirements of WBANs in HCM that includes continuous health monitoring, consistent health data gathering, health data communication over a long distance, low latency, adequate data rate, as well as energy efficiency issue. In addition, the energy consumption issue in WBANs could be traced to its battery-powered sensor devices which depletes their energy quickly during continuous health-monitoring operations when efficient energy-management schemes are not considered [4]. As soon as the power in the sensor devices is exhausted, it becomes difficult for the sensors to communicate vital patients’ health data to remote medical centers. For instance, sensor devices that are implanted in a patient’s body when implementing the WBAN medical application, could be difficult to frequently replace the batteries in the medical sensor devices when they run out of power. Hence, it is essential to design energy-efficient solutions in WBANs for reliable HCM, as well as sustainable network operations. To achieve this complex task, emerging low-power communication technologies are envisaged as an appealing solution that may be explored and employed to achieve energy efficiency in WBANs for HCM, including high health data reliability and a significant reduction in the latency issue. Thus, the major contributions of this research paper are outlined as follows:

- An overview of WBAN systems in HCM, peculiarities, and QoS parameters were thoroughly reviewed.
- The newly emerged LPWAN communication systems were extensively investigated for WBANs in HCM.
- The comparison of the LPWAN communication systems were considered in the context of energy efficiency, security, health data reliability, data rate, latency, cost-effectiveness, and communication coverage, in order to exploit their potentials to support the growth of the next-generation WBAN systems.
- Identification of the power-saving modes and the modulation mechanisms utilized by the LPWAN communication systems to determine their suitability in terms of energy efficiency.
- Discussions and recommendations on the impacts of electromagnetic radiation on the human body.
- Directions for future research to bridge the gaps in knowledge and recommendations are provided.

The rest of this paper is structured as follows: Section 3 presents the overview and concepts of WBANs in HCM including the various types and classification of WBAN medical sensors. Section 4 presents the LPWAN communication systems. Section 5 presents the WBAN QoS requirements. Section 6 identifies the research gaps, open research issues, and recommendations. Section 7 presents the conclusion of this study.

## 2. Related Work

At the moment, there are various survey papers being reported on the legacy short-range communication technologies used in WBANs, while the newly emerged low-power wide-area network (LPWAN) communication systems are yet to be explored. Therefore, we present a survey different from the existing surveys that is centered on the emerging communication systems that could be exploited to achieve a large communication coverage, energy efficiency, and a high data reliability in terms of prompt WBAN health data delivery. It is worth noting that only a few surveys have investigated the usage of LPWAN communication technologies in HCM applications, while its suitability and utilization is yet to be considered at the intersection of WBAN and HCM. For examples of the existing surveys that investigated the legacy short-range data communication technologies used in WBANs, see [5,6,7,8,9,10,11,12,13,14,15]. However, a review of LPWAN technologies for WBAN solutions based on HCM applications does not exist in the literature to the best of the authors’ knowledge. Currently, there are a few existing surveys that investigated the concept of LPWAN communication technologies in HCM which are not based on WBAN solutions and one of such survey is [16]. In [16], the authors discussed the key requirements of the emerging health-care applications and investigated the emerging LPWAN technologies, such as the noncellular-based solutions that include Sigfox, LoRa, Ingenu, and the cellular-based solutions that include long-term evolution (LTE) M1, NB-IoT, and Extended Coverage Global System for Mobile Communication (EC-GSM), with focus on IoT-based personalized health-care applications. They compared the LPWAN communication systems in terms of their energy efficiency and communication coverage. In contrast to the authors of [16], we present a survey on WBAN solutions for remote HCM, exploiting the emerged LPWAN communication systems so as to achieve a prolonged network lifetime, low latency, health data transmission reliability, and real-time WBAN system. In addition, we also introduce the implementation design of the LPWAN solutions in WBAN system architecture. The authors of [17] investigated some LPWAN communication systems, such as LoRa, NB-IoT, and LTE-M, used in hospital environments, but not in the context of WBANs. They focused more on coexistence issues. In contrast to [17], we present a survey on LPWAN solutions that focuses on the network QoS parameters based on energy efficiency, health data transmission reliability, latency, communication coverage, and the electromagnetic radiation issue. In addition, we introduce the LPWAN solutions into the architecture of WBANs. The authors of [18] conducted a survey on the investigation of a WBAN based IoT network for health-care systems. The health-care IoT network architecture, services offered, security, and topology were reviewed; they also briefly identified the specifications of some short-range communication systems, which included 6LoWPAN, Bluetooth, ZigBee, and IEEE 802.15.6. Unlike [18], we present a different survey by introducing the newly emerged proprietary and non-proprietary LPWAN communication solutions for WBAN systems, with a focus on the network requirement factors that includes real-time health data delivery, energy efficiency, communication coverage, and latency for remote HCM. In [19], the authors presented a survey on the application of radio frequency identification (RFID) and ZigBee technologies in IoT health-care environment. Since the authors in [19] did not consider the long-range communication technologies, such as the LPWAN solutions, this provides a context for this new study [19]; this new study exploits the usage of LPWAN solutions in WBAN systems, to improve the remote health-care monitoring of patients’ health conditions, as well as satisfying the network QoS requirements of WBAN systems in terms of enhancing energy efficiency, reliability, and latency performance gains.

For simplicity and clarity purposes, a tabulated survey of the related works with respect to LPWAN communication technologies and LPWAN implementation design for WBANs in HCM is described in Table 1.

Considering the above discussions, it is apparent that most reviews focused mostly on the legacy short-range communication technologies for WBANs, while a few focused on the integration of LPWAN in HCM, such as [16,17], and none has exploited the integration of LPWAN in WBANs for remote HCM in order to satisfy the WBAN communication network QoS requirements, such as energy efficiency, low latency, and health data transmission reliability. Hence, to complement the existing studies in the literature and to address the gap in research, this paper focuses on the exploration and comparison of the emerging LPWAN communication systems, in order to determine the LPWAN technology that is most viable and suitable for WBANs in HCM in terms of low power expenditure, affordability, data rate, reliability of health data delivery, and remote health monitoring of patients. Also, discussions and recommendations on the impacts of electromagnetic waves radiation on the patients’ body were considered. Furthermore, since none of the existing surveys have considered the implementation design of WBANs by using LPWAN solutions with a focus on the QoS requirements of WBANs, this paper considers the integration of LPWAN solutions in WBANs.

## 3. Overview and Concept of WBAN Solutions in HCM

### 3.1. Wireless Body Area Networks

WBAN can be described as a wireless system that consists of serval intelligent and miniature-sized (such as macroscale and microscale) and very tiny (such as nanoscale) sensor devices that can be implanted in a human’s body or worn to continuously or periodically monitor crucial physiological signs, such as the electrocardiogram (ECG), heart rate, blood pressure level, pulse, and temperature, without interfering with their normal personal daily activities. The health data measurements of such sensor devices are sent to a remote medical server, where they can be analyzed for evaluating the health status of patients for decision-making purposes. WBAN solutions could be applied to various other fields, including academics, fitness, industry, military, and sports [20,21]. WBANs can be classified under two main application scenarios, namely, the medical and the nonmedical applications. The WBAN medical applications mainly focus on HCM, which involves the constant measurement of a patient’s health conditions in a seamless and ubiquitous manner, through effective and reliable communication technologies [22]; such applications include ambient assisted living, telemedicine, rehabilitation system, and emergency medical system. Meanwhile, the nonmedical applications are used in the nonmedical scenarios, including entertainment and interactive gaming, sports, and military and defense.

WBAN is a promising technology that can integrate HCM, way of life, and consumer electronics, such as smart phones, around the human body [23]. WBANs are characterized by several interesting promises, including cost-effectiveness, non-intrusiveness, proactive measure, and reliability, as they also offer a lot of benefits to patients, medical experts, and health workers [24,25]. For example, sensors such as an accelerometer can be used to detect patients’ physical activity [26], while a sphygmomanometer (blood-pressure cuff) and insulin-pump can help patients to track and record their physiological vital signs and also assist medical experts, as well as health workers, to remotely monitor and administer treatments to patients. In addition, patients’ emotional reactions could be detected; for instance, in [27], a multi-sensor data-fusion scheme was proposed to automatically detect the handshakes of two people, as well as capture their heart-rate signals based on their emotions, using a collaborative signal processing in node environment (C-SPINE). It is worth mentioning that SPINE is a software programming framework that supports most programmable sensor-device platforms for designing collaborative WBAN applications and networking. For instance, the TinyOS version of SPINE supports the Bluetooth technology, as well as the IEEE 802.15.4 standard, including the implementation of the ZigBee devices [27,28].

**Figure 1:**
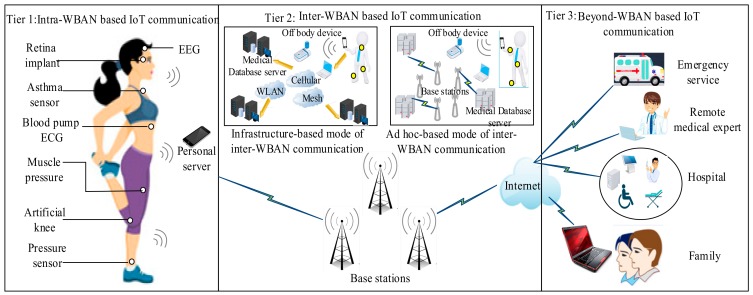
Wireless body area network communication tiers.

Furthermore, as shown in Figure 1, there are three basic communication tiers in the WBAN communication architecture that includes the intra-WBAN communication tier, the inter-WBAN communication tier, and the beyond-WBAN communication tier [29]. The intra-WBAN communication in WBANs can also be referred to as an in-body communication. It is the communication established between the medical sensor devices and a personal server (PS), using short-range radio, such as Bluetooth technology, ZigBee technology, and IEEE 802.15.4 (SPINE project). Consequently, the medical sensor devices in this tier can measure the vital parameters of a patient’s body and forward it to a PS, which acts as a gateway (or a sink node) in the network. The PS can be a smartphone or a cell-phone with internet capabilities, and helps to transfer the measured data received from the medical devices to the next tier, that is, the base station (BS) in the second tier through an internet-enabling communication network or cellular technology, such as 3G, 4G, and 2G GPRS, depending on the nature of the application in terms of the network requirements (such as data rate and power consumption). The inter-WBAN communication depicts the communication between a PS and one or more BS(s). The inter-WBAN communication can be classified into two modes, the infrastructure-based mode and the ad hoc based mode [29], as illustrated in Figure 1. Meanwhile, the communication in the beyond-WBAN is established between the BS(s) and the remote medical centers. To achieve a remote WBAN communication, the inter-WBAN communication tier and the beyond-WBAN communication tier can be linked through a gateway. As an example, a smartphone can be employed as a gateway.

Moreover, since WBANs are typically composed of finite-size medical sensors that have limited energy resource capabilities, small memory space, limited communication, and computation capabilities, then, to efficiently manage a large number of WBANs health data in a large-scale deployment scenario for remote HCM may become a difficult task. Recently, through research efforts, cloud computing has become a promising technology that can offer a large data storage, computation, analysis, and software services at a low cost, when integrated into the WBAN systems. For instance, authors of [30] proposed a BodyCloud Software as a Service (SaaS) method for body sensor networks which can support the development, as well as the large-scale deployment, of cloud-enabled body sensor networks to monitor and analyze the cardiac condition of various individuals in a real-time manner.

### 3.2. Peculiarities of WBANs

WBAN is a unique type of wireless network that is different from the traditional WSNs and other types of networks, such as cognitive radio sensor networks (CRSNs), due to its special features, which include the types of sensor devices to be employed for monitoring, the data rate requirement of the sensor devices to achieve a reliable health data communication, the targeted application environment of the WBAN systems, the traffic pattern of health data, which may either be continuous or periodic, the latency of health data, which requires little or no delay, and the reliability of health data. These unique features of WBAN systems in HCM contribute to the energy issues in WBANs. Note that the success of the requirements of WBAN solutions depend on the energy resources available. Because of the energy efficiency issue in WBANs, power-aware techniques, such as power-efficient communication technology solutions, as well as other methods that can improve energy efficiency, are highly desirable in WBANs for sustainable operations, including meeting the crucial requirements of WBAN systems in HCM. For instance, the authors of [31] proposed an ultra-low power activity recognition system for remote health monitoring, using a metaheuristic optimization scheme that is based on a Grammatical Evolution technique to save energy during data transmission.

## 4. LPWAN Communication Systems in WBANs

Recently, LPWAN solutions are emerging and are gaining recognition over the legacy short-range and cellular communication technologies because of their ability to address long-distance communication, cost-effective connectivity, and large-scale communication at a lower power level. The conventional WBAN systems often employs short-range communication technologies and cellular technologies for their health data communication, which consumes a lot of energy during continuous monitoring of patients’ health conditions because the sensor devices in WBANs mostly rely on battery power. Hence, to achieve the long-standing vision of a long-range communication at a low power cost, real-time data communication, and an energy efficient WBAN system, the LPWAN communication systems are envisioned to be viable for achieving the abovementioned WBAN QoS requirements. Therefore, this research work classifies LPWAN into two major categories, including the proprietary-based communication systems and the non-proprietary-based communication systems. The solutions under the LPWAN categories are discussed as follows.

### 4.1. Proprietary-Based LPWAN Communication Systems

The LPWAN communication systems in this category are also referred to as the noncellular-based LPWAN solutions, which implies they are privately owned technologies that requires proprietary gateways to achieve internet connectivity. The proprietary solutions provide a number of advanced and flexible infrastructure deployment for wireless system communications. As a result of the requirement in the infrastructure deployment, these technologies may not be cost-ineffective. This section therefore explores the types of proprietary-based LPWAN solutions, including RPMA, Sigfox, and LoRa technologies.

#### 4.1.1. RPMA/Ingenu Network

Ingenu technology is a wireless technology that has been in existence since 2008 and was formerly referred to as an On-Ramp Wireless. Ingenu supports a bidirectional communication, that is, the uplink and the downlink communications. This type of technology operates on the 2.4 GHz ISM band and is advantageous when deployed on a free license ISM band compared to it utilization on the sub-1GHz ISM band, because the 2.4 GHz band is available globally, has a wider frequency band, has no duty cycle constraint, and has an additional antenna [32]. RPMA network has a communication coverage of about 3 km in urban regions and could possibly realize a communication link budget of 168 dB, with a receiver sensitivity of –142 dBm and a signal strength that could reach deep in-building, and even underground with a high transmission power [32]. However, there are some drawbacks that could be attributed to the RPMA technology, such as the occurrence of interference that may be experienced during the communication process as a result of the free licensed band it employs. This constraint is likely to affect the usefulness of the RPMA network in WBANs deployed for HCM in terms of energy efficiency, health data reliability, and infrastructure deployment cost.

#### 4.1.2. Sigfox Network

**Figure 2:**
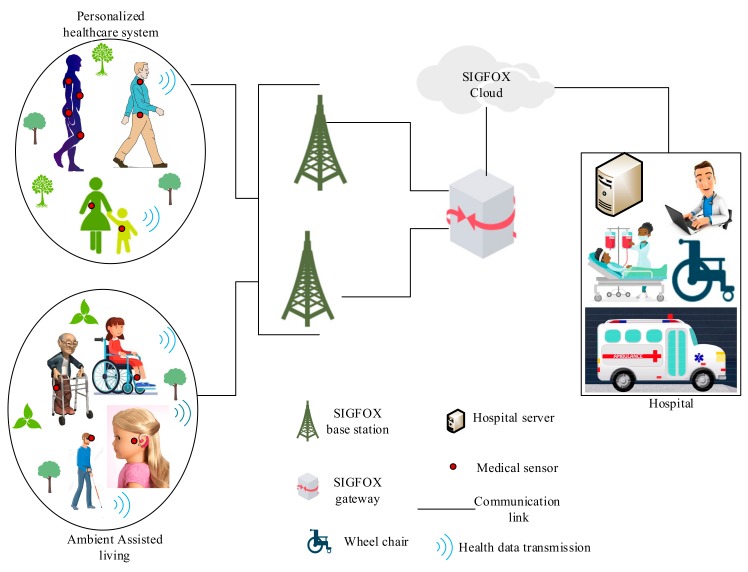
Sigfox wireless body area networks (WBAN) system architecture.

Sigfox is another wireless communication network in the category of the LPWAN proprietary technology that aims to meet the IoT requirements in wireless systems, including WBANs. Sigfox was designed to achieve a long-range communication, easy connectivity, prolonged battery lifetime, better network capacity, and reduced device (i.e., Sigfox module) cost. It offers high QoS requirements which have better resistance to interference issues. Sigfox technology is suitable for low-cost devices that are expected to operate on low power and also need to transmit data across a large communication range. Furthermore, Sigfox utilizes the sub-1 GHz ISM unlicensed frequency band of 915 and 868 MHz in Europe and in the US, respectively, at an extremely low data rate of about 100 bps; thus, Sigfox can achieve high sensitivity over long-range communication by employing a line-of-sight communication method. For instance, Sigfox can attain a communication coverage of around 50 km in rural regions, which can be extended with the help of frequency hopping, while the range reduces to about 10 km in urban regions because of obstructions [33]. With all the mentioned characteristics of this technology, Sigfox can be regarded as an excellent wireless technology that can be used in WBAN medical applications, such as remote HCM to attain low power consumption, long-distance communication, good security mechanisms, and low electromagnetic radiation on patients’ bodies [34] However, Sigfox has a few constraints, including low data-transmission rate, interference issue, and high latency. Also, note that this technology has a limited battery lifespan of about five years, depending on the WBAN application requirements. The architecture of Sigfox communication system in WBAN for health-care application is shown in Figure 2.

#### 4.1.3. LoRa Network

The LoRa Alliance proprietary LPWAN technology is composed of two main components, LoRa and a LoRaWAN protocol [35]. LoRa implies long range and is a patented wireless technology that could be employed in wireless systems, such as WBANs, to target low-powered devices and devices that are not required to send more data at a time. As a result, LoRa could be used to extend the battery lifespan, obtain a low-device cost, achieve a long communication range, and enhance network capacity [36]. LoRa PHY layer operates on the free licensed ISM bands of 868 or 915 MHz, depending on the deployment area. Similarly, a single LoRa BS or gateway could provide coverage in an urban region of about hundreds of kilometers (100 km), depending on the environment and the presence of obstacles. LoRa has a link budget that is higher than any other legacy communication technology standards. In addition, the low-power and the long-range communication characteristics of LoRa makes it an amazing technology for sensing and monitoring systems that includes WBAN systems in HC, as well as WSN systems for environmental monitoring, industrial applications, and smart metering [37].

**Figure 3:**
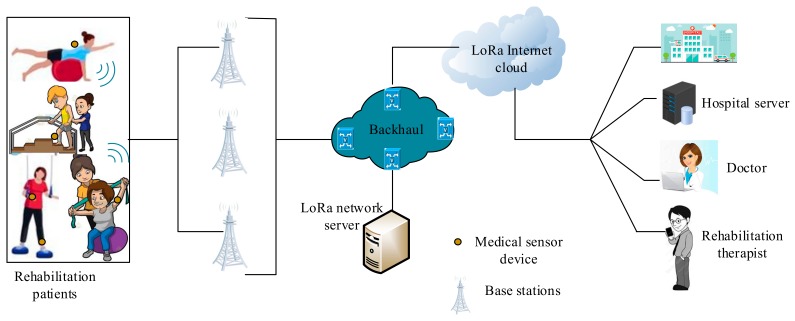
LoRa wireless body area networks (WBAN) system architecture.

LoRaWAN is a wide-area network protocol employed at the MAC layer that incorporates a LoRa technology PHY layer to achieve a network architecture. The protocol utilizes a simple ALOHA modulation mechanism for an uplink communication, using a star network topology to realize a prolonged battery life during a long-range communication process. The papers [38,39,40,41] are the examples of research works that have considered the use of LoRa for HCM. From the aforementioned studies, it can be deduced that a LoRa solution can be potentially incorporated into remote HCM; however, the solution has some drawbacks, such as limited network size, low data transmission rate, high deployment cost, and interference issues. The LoRa architecture, with respect to WBANs, consists of sensor devices, a LoRa gateway, a server, and a remote device that includes a computer device. As shown in Figure 3, the sensor devices are connected to the LoRa gateways and then to the network server through backhaul systems, while the network server is connected via Internet Protocol connectivity, and, from there, the patient’s health data could be retrieved from the hospital database server by the health-care personnel, either to administer treatment or take any further action.

### 4.2. Non-Proprietary-Based LPWAN Communication Systems

The LPWAN communication systems in the non-proprietary category are referred to as cellular-based networks. Examples of the non-proprietary LPWAN solutions include NB-IoT, LTE Cat M1, and EC-GSM IoT, and they constitute the essential building blocks of the emerging 5G network. The 5G technology represents the fifth-generation cellular network technology that is envisaged to have a high data transmission rate, better reliability, and a high bandwidth compared to its predecessors. It is a technology based on the IEEE 802.11ac standard for wideband wireless connections, while the final standard is expected to be set by the ITU [42]. The evolution of the 5G technology is becoming the major advancement of IoT applications in terms of popularity. As a consequence, the 5G cellular technology would contribute greatly to the next generation of WBAN systems through the massive connection of billions of intelligent objects, such as sensor devices, to achieve an amazing ecosystem.

It is envisioned that the 5G technology should be able to transmit at a data rate of about 10,000 Mbps in urban areas and 1000 Mbps in rural areas. The 5G technology would integrate a transport network requirement in its architecture to improve sustainability in data communication by employing a self-backhauling technique [43]. Moreover, it is also envisioned that the 5G should be able to provide a wider bandwidth of more than 100 MHz, with frequencies ranging from 3 to 5 GHz. Furthermore, the 5G technology is also expected to provide a fast, flexible, and reliable network, and this would help WBANs to achieve better remote monitoring of patients’ health conditions. It is also expected to encourage the speedy transmission of data and videos, and this may be advantageous in the area of telemedicine, to assist surgeons to perform operations by using robotic scalpel.

The non-proprietary LPWAN solutions employ the existing cellular network infrastructures for internet connectivity compared to the proprietary LPWAN solutions. As a consequence, the technologies in this category could be considered cost-effective in terms of infrastructure deployment cost. The solution technologies identified in this category are discussed as follows.

#### 4.2.1. LTE CAT Technology

The LTE Cat technology is also referred to as an enhanced machine-type communication (eMTEC), as well as LTE Cat M or LTE-M in wireless networks, including WBAN systems. This technology could be regarded as an appealing and promising LPWAN solution, as proposed by the 3GPP in the Release 13 standard to achieve low power consumption, extend communication range, minimize deployment cost, and low complexity. The LTE-M adopts a new power-saving scheme and an extended discontinuous reception (eDRX) scheme to prolong the battery lifetime of the LTE-M-enabled devices to more than 10 years. Also, the LTE-M can achieve a peak data rate of 1 Mbps for uplink and about 384 kbps for downlink communications, with a latency that ranges between 50 and 100 ms. LTE-M can achieve an excellent communication coverage of about 11 km, with a maximum throughput that is less than or equal to 1 Mbps. Moreover, LTE-M could be employed in a plethora of IoT use cases, including WBANs in HCM (such as patient monitoring and wearables), security systems (i.e., home security monitoring), industrial applications (such as assets management), and transportation [44,45]. However, LTE-M has limited communication coverage which could only support about 20,000 devices on a BS.

#### 4.2.2. EC-GSM Technology

**Figure 4:**
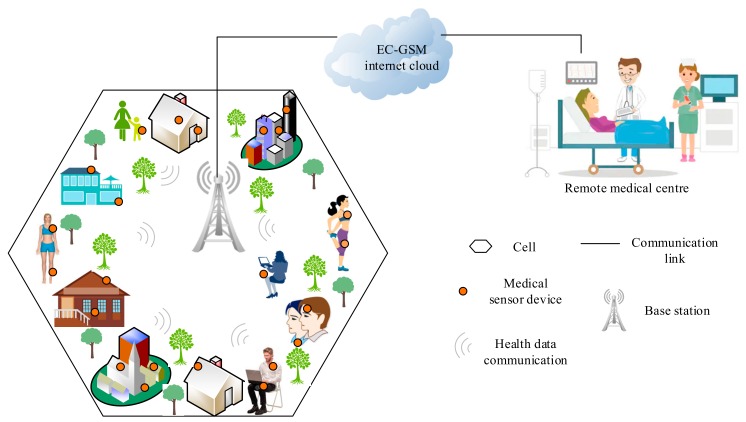
Extended Coverage Global System for Mobile Communication (EC-GSM) cell communication coverage to remote medical center in WBANs.

In EC-GSM technology, the 3GPP proposed an extension of the GSM coverage by adapting and leveraging the legacy 2G infrastructure in order to offer a reliable and efficient IoT connectivity in wireless networks. This technology employs two modulation schemes that include the 8-ary phase shift keying (8PSK), which provides support for a transmission rate of about 240 kbps and a Gaussian Minimum Shift Keying (GMSK) with a data rate of about 350 bps to 70 kbps. In addition, the EC-GSM communication network target about 50,000 sensor devices in a single cell and may support a link budget within the range of 154–164 dB based on the power of transmission; it also employs an eDRX modulation mechanism to enhance energy efficiency [46,47]. For clarity purposes, it is noteworthy that a cell is the geographical area that a single BS can service (or cover) in cellular networks, and a typical scenario of the EC-GSM technology in the context of WBAN domain, including health data transmission to a remote hospital, is provided in Figure 4.

#### 4.2.3. NB-IoT Technology

NB-IoT technology is also known as the LTE Cat-NB1. This technology is a promising standard in the 3GPP specification that was designed to achieve low-power communication, in order to extend the battery lifetime of sensor devices, improve signal coverage, and also enhance flexible deployment. Furthermore, NB-IoT technology utilizes the existing cellular technology infrastructures (such as cellular mast) which helps to reduce its deployment cost, and it is supported by over 30 mobile network operators worldwide, which offer communication coverage for about 3.4 billion devices and are geographically capable of providing services for over 90% of IoT use cases, such as WBANs. NB-IoT could be employed in a variety of IoT applications, such as wireless networks that include WSNs, WBANs such as eHealth, smart tracking and metering, emerging industries such as smart city, and smart agriculture [48].

Moreover, NB-IoT could coexist with the legacy GPRS, LTE, and GSM technologies and attain an excellent performance. At the MAC layer, the NB-IoT technology can achieve an uplink transmission of a data rate that ranges from 0.3 to 180 kbps with a single carrier-frequency division multiple access (SC-FDMA) technique, while, in the downlink communication, it may transmit at a data rate within 0.5–200 kbps and use an OFDMA modulation technique for its downlink transmissions at the PHY layer [49]. Similarly, NB-IoT could achieve a latency of less than 10 s for an uplink data transmission and can provide a communication distance of about 35 km [38]. NB-IoT devices could support a transmission power of about 20–23 dBm, and the battery lifespan of the NB-IoT devices could be extended to more than 10 years by exploiting battery-power-saving techniques, including a power-saving scheme, as well as an eDRX to achieve energy efficiency. The exploitation of the power-saving scheme helps to optimize power consumption, by allowing sensor devices to enter a sleep mode when not in use, while eDRX is used to extend the sleeping cycles of a system’s idle modes.

**Figure 5:**
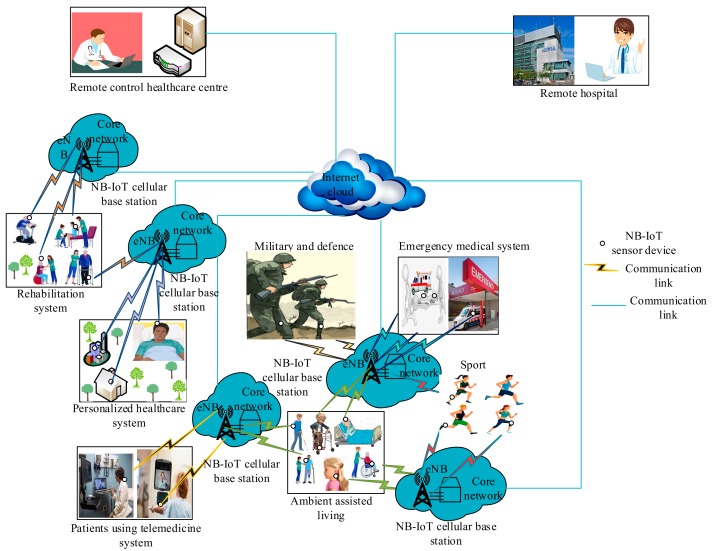
NB-IoT communication for WBANs in HCM.

The NB-IoT architecture for WBANs, as shown in Figure 5, comprises the NB-IoT medical sensor devices, NB-IoT BS (s), an internet cloud platform, different WBAN-application scenarios, and remote health-care centers. The medical sensor devices are employed to transmit health data to the NB-IoT cellular BS (s), and, thereafter, the BS (s) conveys the health data to an internet cloud platform, where the health data would then be forwarded to the remote health-care centers. Note that the NB-IoT medical sensor devices are connected to the internet cloud via an evolved node B (eNB) since they do not require a gateway to connect to the internet cloud platform, as they are mounted on the existing cellular infrastructure technologies. Considering the capabilities of the NB-IoT technology, it promises to advance next-generation WBANs in HCM in terms of meeting crucial WBAN QoS requirements, including high energy efficiency, long-range communications, the ability to support many sensor devices, and the high data reliability support. For example, the authors in [50] proposed an architecture using NB-IoT to connect intelligent objects in a smart hospital environment. Another study [51] investigated the performance of NB-IoT in health care, while NB-IoT challenges and issues are discussed in [52,53].

### 4.3. Summary of LPWAN Technologies

Since WBANs health data are critical in nature and require little or no delay in communication, the LPWAN solutions are proposed as promising solutions, and it is apparent that they have the capability of playing important roles in WBAN systems, especially in terms of health delivery reliability, power efficiency, and long-distance communication coverage to remote health centers in a seamless manner. In addition, unlike other communication solutions, such as ZigBee, Bluetooth, and the traditional cellular networks, the battery lifetime of the LPWAN based sensor devices could last for more than 5 years, subject to WBANs’ use cases [54], which is quite reasonable for the WBAN systems. In addition, in terms of communication distance, LPWAN solutions can provide support for a large communication coverage that ranges from 10 to 40 km in rural regions and 1 to 5 km in urban regions [55]. In terms of satisfying the critical WBANs’ requirements, it is important to point out that each piece of LPWAN technology has its own benefits and costs; for example, NB-IoT could provide more bandwidth, while LoRa may be more energy efficient. This indicates that NB-IoT has better data-handling capability when compared to LoRa. For comparative purposes, Table 2 presents the summary of different LPWAN solutions that could be employed in WBAN systems. It is imperative to emphasize that the choice of communication technologies to be integrated into WBAN systems needs to holistically consider several factors, including low electromagnetic radiation, since WBANs are human-body-focused networks. Also, data rate, deployment cost, deployment infrastructure flexibility, and health data delivery reliability should be put into consideration, so as to satisfy the WBAN QoS requirements.

## 5. WBAN QoS Requirements

To be able to effectively and continuously connect and send information about patients’ health status to medical personnel in remote locations, WBAN systems are expected to satisfy some stringent QoS requirements. The following are some of the most important requirements to be considered when developing a WBAN system in terms of efficiency and reliability.

### 5.1. Energy Efficiency

Energy efficiency can be described as a design consideration used to minimize or reduce energy consumption in a system. Energy efficiency techniques can be employed in WBAN applications, to achieve a sustainable WBAN system. The following are the major reasons why energy efficiency is very important in a WBAN system:

- Medical personnel are often situated far away from their patients, especially patients that are suffering from chronic diseases, so the only cost-effective way that could be employed to monitor such patients is by deploying efficient HCM sensing and communication technologies to gather and transfer medical data about such patients. The sensor devices involved in the technologies cannot afford to spend more energy on data gathering and data transmission since they mostly operate on batteries.
- Energy efficiency is a fundamental issue in any communication network. Once the energy of a network is exhausted or drained, the network becomes inactive. Therefore, it is very necessary for a WBAN system to be energy efficient in order to achieve a sustainable network operation.
- Due to the disparity in the WBAN applications, some applications require low data rate, while some require high data rate. The disparity in data rate requirements among WBAN applications will encourage a higher amount of power to be consumed by the applications with high data rate requirements. This is technically due to the trade-off in data rate and power consumption. Therefore, to strike a balance between power consumption and high data rate, there is a need for energy-efficient schemes to optimize data transmission, in order to reduce energy spent on data transfer.

### 5.2. Health Data Security Requirement

Another very important requirement is the security of WBANs health data, and this can be directed toward privacy, confidentiality, data encryption, and integrity. Security in WBANs could be referred to as the protection of a patient’s medical data or information from any unauthorized persons when the data is being collected, processed, transferred, or stored. In the context of privacy and confidentiality, patients’ health data must be protected in such a way that only authorized medical personnel have access to the collection, as well as the usage, of patients’ health data and also ensures that their health information is not divulged to any third party or eavesdropped [59]. If false or wrong data is given to a doctor as a result of the system being compromised, it may cause the wrong treatments and prescriptions to be administered, and this may result in a premature death of a patient. Data integrity and encryption simply implies protecting the content of a patient’s information. An encrypted WBAN must be able to identify if an information is sent from an imposter or from a well-known, trusted medical center to a patient during health monitoring.

### 5.3. Health Data Transmission and Reliability

Data rate could be referred to as the amount of health data and also the speed (or rate) at which the health data can be transferred over a communication link at a specific time. In WBANs, data rate requirement differs depending on the nature of an application and the type of the application traffic pattern, such a continuous traffic and periodic traffic. Generally, a WBAN sensor device requires a data rate of a few kbps (<1 kbps) for on-body monitoring to a very high kbps (>1000 kbps) for real-time video streams [60]. Similarly, for health data transmission to be reliable, it is necessary to ensure optimal bit error rates (BERs) when considering the design of WBAN systems. The BER could be described as a measure of the number of bit errors per unit time (data lost). For example, to satisfy the reliability requirements of health data transmission in WBANs, the sensor devices with a high data rate requirement may be operated in environments that are characterized by a less value of BER, for example, ten raised to a power minus ten, while the sensor devices with a less data rate requirement may be operated in environments characterized by a high value of BER such as ten raised to a power of minus four [61]. Similarly, by exploiting different coding and adaptive modulation techniques that are appropriate for the communication-channel conditions at the PHY layer of a WBAN system could be used to optimize the BER value of a transmission link [62].

### 5.4. Health Data Latency

Latency is referred to as the amount of time (or delay) it takes data to travel from one device to another. To avoid delay in the delivery of health data to remote health-care centers, the latency for medical applications should be minimized in WBAN systems. For example, in medical applications, the latency of health data should not be up to 125 ms, while a data latency that is not up to 250 ms should be ensured in nonmedical applications [63]. The strictness in the data latency requirement of WBANs is due to the fact that most medical applications, for example, emergency medical systems, cannot tolerate low response times because if the gathered health data is not delivered in time to a health-care center it may become useless when dealing with emergency situations. However, it is noteworthy that a WBAN system may encounter delay issues in health data communications as a result of interference challenges if the employed communication technologies operate in overcrowded bands [64]. To handle latency in health-care applications, the authors of [65,66] highlighted a few solutions that may be useful in WBAN systems.

### 5.5. Throughput Requirement

Throughput is used to measure the unit of data a system can process in a given period of time. It could further be described as the total amount of data that can be transmitted over a communication channel bandwidth by a WBAN system. Since most WBANs health data are critical and require timely delivery of the health data to a remote medical center, an optimum throughput is needed to enhance the health data communication efficiency of WBAN systems [67]. To achieve this, the throughput of a WBAN system can be optimized either by maximizing the individual throughput of a senor device in a WBAN network or the overall network throughput which implies all the sensor devices.

### 5.6. Coexistence Issues in Communication Bands

WBANs can be described as health data monitoring and communication systems. As a consequence, WBANs being communication systems requires a communication band to provide communication channel(s) among the network devices. For more clarity, a communication band may be referred to as a frequency band. It is a given range of frequencies in the radio frequency (RF) spectrum. For economic reasons, WBANs are mostly designed to function in the unlicensed industrial, scientific, and medical (ISM) frequency bands, such as 2.45 GHz, including the 915 MHz. For instance, communication technologies, including ZigBee, Bluetooth, Wi-Fi and some other standards, which are often employed in WBANs for health data communication, operate on the ISM bands. Note that other communication systems, apart from WBANs (for example, WSNs and cognitive radio networks (CRNs)) also operate on the ISM bands. Consequently, the number and diversity of these wireless communication technologies are constantly increasing and making the band overcrowded. Due to the overcrowded nature of the ISM bands, the plethora of communication systems that coexists on the bands may suffer from interference. Thus, to make WBAN medical applications reliable especially in emergency situations, there is a need to implement techniques that have resistance to interference.

### 5.7. Low Electromagnetic Radiation

High electromagnetic wave radiation could be very dangerous to human’s health because of its potentials to cause different health hazards, such as tissue destruction, lower blood flow, and enzymatic disorder [68]. Since WBAN is a body-focused network i.e., a network that has direct contact with a patients’ body, it is therefore essential to put into consideration the electromagnetic waves that are radiated by the sensor devices during data communication when designing a WBAN system. Also, it is noteworthy to mention that a body sensor device that transmits at a high-power level will achieve a high throughput, which is quite good, as higher throughput rates enhances the packet success rate for a successful delivery of health data. However, transmitting at higher power levels by the sensor devices will automatically increase the electromagnetic radiation absorbed by the patient’s body tissue, and this will also go against the acceptable specific absorption rate (SAR) of body tissue for 1 g at 1.6 W/kg, as defined by the Federal Communications Commission (FCC) regulation. SAR is used to measure how the human body and its tissue absorbs energy or RF energy from electromagnetic radiations when exposed to an RF electromagnetic radiation. To satisfy the SAR constraint requirement, each medical sensor device in a WBAN system is expected to radiate its health data at a transmission power of about 0.0001 W, which is equivalent to –10 dBm and should not exceed a maximum transmission power <0.001 W or 0 dBm.

### 5.8. Mobility Support

Patients’ mobility support is another crucial requirement in the WBAN systems for providing ubiquitous HCM services. Since most patients who wear WBAN are not likely to be static in nature but, rather, mobile users that need to access and receive medical services at their convenient different locations such as at home, office, market, and so on. Hence, patients’ slow or fast movements could result in signal fading or shadowing that may be unfavorable to the reliability, as well as the QoS performance, which includes health data delivery ratio, latency, and so on. Therefore, the current WBAN solutions needs to incorporate some wireless technologies into the systems in order to address the issue of patients’ mobility. For this reason, the LPWAN solutions are envisaged as promising technologies that can provide mobility to patients and support large distributions of health data to remote medical centers. For instance, a patient’s temperature or heart rate can be monitored anywhere through temperature or pulse rate medical sensors and notify the medical expert in charge about the health condition of a patient when connected to the LPWAN systems.

### 5.9. Interference Issues

Interference is a serious problem that has the tendency of affecting the productivity of a network (such as WBANs) in the areas of energy efficiency, latency, throughput, and so on. Since the WBAN systems may coexist with other communication networks such as the CRSNs, WSNs, IEEE 802.15.4, IEEE 802.11, and the traditional cellular networks, that is, they operate on the same 2.4 GHz unlicensed ISM band with the WBAN systems, co-channel interference may be experienced in the WBAN systems. Additionally, mutual interference within the WBAN systems could occur, too. Mutual interference in the WBAN systems is mainly caused when medical sensors transmit their health data simultaneously with other neighboring WBANs [69]. As a consequence, the interference problem may result to energy inefficiency (i.e., high energy consumption), low throughput, and high latency due to signal distortion; for example, the energy required to send 10,000 bits of health data could only be used to send 1000 health data bits successfully due to the frequent retransmission process that occurs when the wireless systems on this same frequency band with the WBAN systems sends their data concurrently, resulting in interference problems. For this reason, the medical sensor battery that was meant to last for a year will only last for a month as a result of the high energy consumption problem that may be experienced in the network due to the interference issue. In addition, the network becomes unreliable, which may lead to high mortality rate, since WBANs health data are critical in nature and require real-time delivery, especially in emergency situations. Therefore, issues like interference should be considered when designing a WBAN system for remote HCM. In Table 3, a brief summary of some of the QoS requirements for both medical and nonmedical applications in HCM in relation to the sensor types, data rate, frequency, accuracy, energy consumption, and latency is presented for simplicity’s sake.

## 6. Research Gaps and Recommendations for Next-Generation WBANs in HCM

The challenges of the traditional HCM systems have necessitated the need for more advanced, reliable, low-cost, and efficient health-care systems. In the quest to address the challenges associated with the traditional HCM systems, a context was provided for WBAN solutions. Unfortunately, the existing solutions of WBANs in HCM are constrained by several issues, such as limited energy resources, high deployment costs, latency, and reliability issues. Most of these issues are caused by the standard communication systems currently employed by the present generation of WBAN systems. To help WBANs achieve their promises of effective, efficient, reliable, and low-cost monitoring of patients’ health conditions, research efforts are currently ongoing in academia, as well as in the industry. To address some of the issues in WBANs, note that wireless communication technologies are capable of playing imperative roles for achieving new strategies that could be exploited for improving WBAN solutions in HCM. Interestingly, recent advances in communication systems are envisaged to provide new strategies to improve WBANs, as well as provide support for remote patients’ health monitoring and treatments in a reliable and efficient manner in the next generation of WBANs.

However, it is obvious from the existing solutions on WBANs that most works adopt the short- and medium-range communication technologies, while the possible opportunities of the emerging LPWAN solutions in the context of energy efficiency, health data delivery reliability, low latency, and long-range communication are yet to be explored. As a consequence, this study provides insights into the exploitation of the LPWAN communication systems for the realization of next-generation of WBAN solutions in HCM to achieve energy efficiency, long communication range, low latency, and health data delivery reliability. With the incorporation of the emerging LPWAN communication systems into WBANs, the early detection and treatment of diseases, especially chronic diseases such as cancer, cardiovascular, Parkinson, and congenital heart diseases, could be achieved for real-time monitoring of patients living with any of the diseases mentioned above, including other diseases, thereby helping to reduce mortality rate. Furthermore, for the dreams of WBAN systems to come true, outstanding recommendations are provided as follows.

### 6.1. Development of Efficient Transmission Strategies for Improving LPWAN-Enabled WBAN Communication Systems

The development of efficient transmission optimization schemes is highly desirable for LPWAN enabled WBAN systems. Such schemes are important because there is a great need to optimize the transmission power of WBAN sensor devices due to the thermal effect of higher transmission power on the human body and its tissue with greatly damaging impacts. For example, authors of [76] addressed the thermal rise problem, as well as the QoS provisioning issue, for the intra-WBAN communication by designing a thermal-aware QoS routing protocol for in vivo sensors in WBAN systems. It is noteworthy to mention that the development of efficient transmission optimization schemes should not only focus on the optimization of the transmission power of the WBAN sensor devices, but should holistically cater for the regulation of the absorption of electromagnetic radiation from the electromagnetic waves that are radiated by the sensor devices in WBAN systems. To achieve this, a Markov Decision Process (MDP) technique can be potentially exploited to optimize the transmission power of WBAN sensor devices during health data communication, to reduce energy resource consumption and to minimize the thermal effect of the electromagnetic radiation at the transmitter side of the WBAN sensor devices, to lower the SAR of the emitted waves in order to arrest its negative impacts on the human body and its tissues. This can be realized by setting an SAR limit that must not be exceeded by the transmission power of each sensor device, and such transmission power should not compromise the achievable throughput performance of the sensor devices. Since there is a trade-off between the transmission power and the achievable throughput of a sensor device due to the fact that high-transmission power will increase the throughput, then optimal transmission schemes should be sought for a successful delivery of health data. Research on designing transmission policies for improving communications using MDP was done [77] for intra-BAN communication. The proposed solution was designed offline by employing an iteration method which was integrated into the nodes to be reflected when making routing decisions. Another significant aspect of this work is the ability to restrict heat generation. The reliability of the approach was evaluated in terms of packet delivery ratio; the work could still be extended by considering more performance parameters, such as the energy efficiency, system throughput, and delay time of the network.

### 6.2. Improving the Efficiency of the Medical Nanosensor Devices in WBAN Systems

Recently, nanotechnology was applied to the domain of the health-care systems, to improve the quality of health-condition monitoring, and this has brought medical nanodevices such as the nanosensors into existence in the field of WBANs. The medical nanosensors are portable sensor devices that can hardly be seen with the naked eye, as compared to the traditional medical macro sensor and microsensor devices used in the health-care systems to monitor patients’ health conditions. There are several benefits that could be attributed to the medical nanosensors employed in the medical scenario, such as a reduced size, which makes such sensors easier to wear in or around the body, improved real-time monitoring and response time, high sensitivity, and low-cost. However, the problems of the medical nanosensors are more complex, unlike those of the medical macro sensor and microsensors. For example, communication and power issues are complex research problems in the medical nanosensor devices because of their very tiny size, which has constrained them with a very tiny nano-communication radio and a very tiny nano-battery power source with limited nano-power. These constraints have posed more complex research challenges to be tackled in WBAN systems that are coupled with the medical nanosensor devices. Such challenges and insights on how they can be addressed are as followed:

- How the nanosensors can effectively communicate their data because of the tiny nature of the allowable radio, i.e., how the communication channel of the medical nanosensors can be efficiently modelled. For communication purposes, two major communication alternatives are envisaged, the nano-electromagnetic communication and the molecular communication. The molecular communication has been proposed in recent times as a newly emerging communication paradigm that employs biochemical signals to transmit information from a nanodevice to another within a short distance [78]. More research on the channel modelling and the development of networking protocols could be investigated and exploited for the nanosensors in WBAN systems. The electromagnetic nanosensors employ electromagnetic radiation to transmit information. It can support two frequency bands for their operation, such as the megahertz band upper part and the terahertz band (0.1–10 THz). The megahertz frequency could be enabled by employing an electromechanical nano-transceiver, while the terahertz band could be achieved through novel nano-antennas. For instance, a research work was done [79] for intra-body nanoscale communication, in which the authors developed a robust terahertz channel model for in-body communication, taking into consideration the impact of the propagation of the electromagnetic waves and the molecular absorption generated from the patients’ tissue. More research to insightfully investigate the accuracy of this research can be done and also developing new channel models for the terahertz band for the WBAN communication systems. In addition, more research investigations into developing novel health information encoding and modulation methods that can encourage the exploitation of the wide bandwidth offered by the terahertz channel are worth exploring. Additionally, novel communication protocols for the electromagnetic wireless nanosensor networks could be explored for further research.
- How to efficiently utilize the ultra-limited power resources also calls for a serious concern for a fruitful WBAN system deployment. To address this issue in the nanosensor-based WBAN systems, since such systems cannot afford to spend much power to transmit their health data due to their ultra-limited power constraint, new policies for optimal power control can be pursued by using energy-efficient techniques, like network coding, optimal control methods, queuing theory, and game theoretic methods.
- The placement of the medical nanosensor devices among multiple alternatives is also an interesting problem that needs to be deeply investigated, as this would enhance the power efficiency of the WBAN systems since power constraint is a complex issue in WBAN systems that are coupled with medical nanosensor devices. As an insight into addressing such problem, the consideration of new optimization algorithms for the optimal placement of the devices is envisaged as a promising solution.

### 6.3. Development of Energy-Aware MAC Protocols for WBAN Communication

Since the energy-efficiency issue is a major concern in most wireless networks, such as WBANs, developing energy-efficient techniques, such as power-aware MAC protocols, is highly desirable for effective and efficient WBAN communication systems. It is important to point out that the MAC layer is one of the most crucial layers and needs more attention in the context of energy efficiency, due to the fact that it is the layer responsible for the handling and controlling of the transmission power, and, at the same time, it is the layer where major causes of power wastage issues like retransmission, collision, carrier sensing, and idle listening are experienced. In recent times, different MAC protocols have been developed and proposed for the MAC layer, but most researchers have focused more on improving the bandwidth utilization, throughput, and latency concerns of the MAC protocols, while only a few consider energy-saving mechanisms, such as energy-aware scheduling methods, directional antenna, clustering approach, wake-up schemes, and so on, to reduce energy wastage in the WBAN system during communication. Moreover, there is still much room for improvements, as the current literature on energy aware MAC protocols for WBAN systems is yet to fully address the energy-efficiency issue; for example, the newly emerging communication systems require new novel MAC protocols to be studied for them to be well adapted into WBAN systems to improve their efficiency when it comes to things like energy utilization and delay. To address the MAC protocols’ energy-wastage issues, efficient algorithms that can adaptively, as well as asymmetrically, manage the power balance between the medical sensor devices and the coordinator (BS) could be explored by researchers through the use of optimal control methods, like game theory, fuzzy logic, and so on. Additionally, further improvements on the reduction of routing redundancy and overhead traffic control to achieve energy efficiency are also promising research areas that require more attention.

### 6.4. Employing Software Defined Nework (SDN) Technology to Improve WBAN Energy Efficiency

SDN has recently emerged as a promising networking paradigm that can be coupled with WBAN systems to improve energy efficiency due to its capability to act as a centralized control system (or controller) to control the intelligence of the WBAN systems and to optimize the usage of the system’s resources. However, the SDN is currently crippled by a number of open research issues relating to the performance, reliability, and security, which are critical QoS requirements in WBANs. The issues are caused by the controller centralization and call for more research investigations to make SDN useful in the WBAN systems in terms of improving the performance, reliability, and the security efficiency. Because of the issues resulting from the controller centralization, a possible useful idea is to fragment the control system by using a distributed method.

### 6.5. Addressing Energy-Efficiency Issue in LPWAN-Enabled WBANs through Congestion Control Using Queuing Theory

In a LPWAN-enabled WBAN communication system, WBAN sensor devices may potentially encounter a congestion issue, especially during a many-to-one health data communication when a protocol such as the nonorthogonal multiple access (NOMA) mechanism is used as a medium for health data communication, as well as when the available channel capacity is exceeded by a heavy load or offered traffic load at a specific period of time. Such issues have the tendency to negatively impact the energy efficiency performance of the WBAN systems when efficient network traffic control mechanisms are not implemented in the network. It is important to emphasize that energy efficiency is a critical WBAN performance measure due to the constrained resources of the medical sensor devices, such as energy, and the congestion issue is also a critical problem in communication systems in general. For example, a work was carried out [80] to address energy consumption and congestion-related problems in WSN. The significant part of the work is the ability to detect the network congestion effectively, and the proposed solution was obtained through the modelling of the network into an equivalent queuing model network. Another research work was carried out [81] that used a rate-control mechanism for mitigating congestion in WBANs. The authors of [82,83] proposed a fuzzy logic congestion-control scheme with a priority-based rate for WBANs. The proposed system in [82] is capable of differentiating patients’ physiological signs and prioritize them according to their importance. While, in [83], the solution presented was capable of monitoring patients’ health conditions and transferring their health data to a remote data center. Moreover, researcher may look into addressing the trade-off between the energy efficiency and the congestion issue in the LPWAN enabled WBAN systems by studying and exploiting the queuing models such as the M/M/1 queue model, priority queue model, M/G/1 queue model, ∑ MMPP/D/1/S, Geo/G/1, and ND policy, as well as the hybridization of the Geo/G/1 and the ND policy queues as a queue-assisted congestion controller to improve the energy efficiency of the LPWAN enabled WBAN systems.

### 6.6. Consideration of Interference Management Solutions in LPWAN-enabled WBAN Systems

Interference can be described as an unwanted signal which modifies a useful signal in a disruptive way. It is very important to point out that addressing the co-channel interference issue within a network is much easier than in the whole communication network system because of issues like the different MAC protocols employed, different bandwidth, data size, and different transmission power employed. Considering interference within the WBAN systems for the inter-WBAN systems to function properly the IEEE 802.15.6 task group transmission range specification for 10 WBAN systems that are jointly located is about 3 m apart [84]. Then, a WBAN system is likely to encounter other neighboring WBANs; for instance, interference may occur when several patients wearing the WBAN sensor devices step into the range of one another and then coordination may become impractical. The incoordination difficulty is due to patients’ unpredictable postural movements that may lead to network moving in and out of their range. Therefore, interference problem can be addressed either within the WBAN system or WBANs with other communication networks in the unlicensed spectrum by developing efficient interference-management schemes. For instance, the authors of [85] used the spectrum-splitting approach, which suggested that spectrum could be divided into two parts: the first part would be dedicated to the device-to-device users, and the second part to the cellular users. Such spectrum-splitting strategy could also be adapted to manage interference between WBANs and other communication networks. Note that the spectrum-splitting strategy could only be used to address the interference between WBANs and other communication networks; consequently, an additional scheme for within the WBAN systems interference management is needed in the spectrum-splitting approach, and this poses an open research issue. Another method that can be used to mitigate interference within WBANs is the employment of a mode-selection algorithm. Mode selection may be performed based on path loss, the distance between the sensors, and the signal strength received over the sensor-to-sensor link. Furthermore, research investigations can be carried out on using radio resource allocation to tackle issues like how to allocate frequency resources in an optimal manner to a group or paired medical sensor devices in order to optimize some QoS performance parameters. Additionally, techniques like time-frequency hopping could be used to reduce interference between the WBAN system, as well as WBANs and other communication systems [86].

### 6.7. Improving WBAN Mobility Support

At the moment, there is a lot of researches that has considered the mobility support issue and has designed new networking models for WBANs, but it is worthy to still highlight some points for further investigations, so as to improve the WBAN QoS requirement via prompt health data delivery, real-time health monitoring, and latency. In order to improve WBAN mobility support, the following areas can be explored.

#### 6.7.1. Mobility Predictions and Management

The management of mobility support for WBAN is very crucial and still needs much room for further study, especially for ubiquitous health-care monitoring; for instance, critical health data should be routed in a seamless manner among the mobile WBAN sensor devices, without link disconnections and in an unobstructed way. WBAN mobility management is very important in dynamic environments where there are network topology changes, and thus the mobility coexistence of WBANs could affect their productivity. However, there are several investigations on mobility predictions for WBANs [87,88]. In [87], a mobility prediction based on an optimal control handover mechanism for WBANs was proposed by employing stable routes, as well as a hop-by-hop policy, to get to the BS in a dynamic setting. The authors of [88] proposed a prediction secure and routing (PSR) framework that is based on regular body movements, which can predict topology changes and guarantee connection quality. In recent times, smartphones have become a widespread technology, so the use of smartphones to predict patients’ mobility pattern could also be explored.

#### 6.7.2. Mobility Models

Mobility models are usually designed to describe patients’ pattern of movements, as well as their changes in location, based on direction and velocity over a period of time. It is therefore essential for the WBAN mobility models to emulate the pattern of movement of targeted real-life application scenarios. However, most WBAN research work is based on performance evaluation, which is used to prove their simulation results. Mobility models are very crucial in performance evaluation, meaning that mobility models should be designed in a cooperative way by employing a network simulator. The authors of [89], for example, proposed a mobility model for simulating the intra-WBAN protocol and the extra-WBAN protocol. The model is a configurable mobility model and is implemented as an add-on to the OMNeT++ mobility framework. It is noteworthy to mention that OMNeT++ and Network Simulator-(NS) 2/3 are generally used as frameworks to conduct WBAN mobility simulations; hence, there is a need to get a WBAN mobility model that is free and available as an add-on to these frameworks. In order to meet this need, a mobility scenario-generating and analysis tools are envisaged as promising tools for the mobility model; an example is the BonnMotion software [90]. Consequently, further analysis could be done to ascertain the mobility model accuracy. Although, a few applications are recently deployed and available now, the existing mobility models are still not enough to accurately describe the sensor nodes’ movement in patients; therefore, more research on patients’ behavior is highly necessary in order to improve the mobility models. Additionally, since patients’ behavior could be affected by various conditions, like where they live and their age, these conditions should also be put into consideration when designing a good mobility-support model for WBANs. More research work could also be done on the existing WBAN mobility models so that they can cater to patients’ environments, including city roads for walking, jogging postures, running postures and so on, thus combining different mobility models, which explains the movement boundary that is envisaged to improve the mobility model accuracy; an example is the Manhattan model.

### 6.8. Development of Energy-Efficient Resource-Allocation Schemes to Enhance the LPWAN-enabled WBAN Sensor’s Lifetime

Different energy-efficient resource-allocation schemes should be explored for potential exploitations in the LPWAN-enabled WBAN solutions so as to optimize the scarce network resources that include energy, time-slot in communication channels, and spectrum. Also, it is noteworthy that, during the allocation of these network resources, there should be fairness in the allocation of the resources, to improve the energy efficiency of WBANs deployed for HCM. Some energy-efficient resource techniques, including the sleep/wake-up schemes, as well as energy-efficient routing protocols with tools like queuing theory, fuzzy logic, and game theory, could be used to allocate energy resources in the LPWAN enabled WBAN. For instance, a queue sleep/awake approach was analyzed by using the min (N, T) strategy in terms of an M/G/1 queue to mitigate energy utilization of a sensor node in [91]. Also, a fuzzy logic based dynamic time slot allocation mechanism was proposed for a fog-enabled network to monitor patients remotely in [92]. The proposed scheme was capable of eliminating the time-slot wastage in the network. Another research work [93] proposed a coalition game-based approach to maximize the system throughput. However, future research on energy efficient resource allocation could be carried out based on intelligent policies, by employing artificial intelligence techniques, such as fuzzy models, neural networks, and machine learning.

## 7. Conclusions

In order to enable WBAN solutions to achieve the vision of a sustainable remote HCM, it is important to tackle the drawbacks of the current WBAN solutions for HCM applications. Such drawbacks include energy efficiency and reliability of health data delivery to remote health-care centers, which could be attributed to the short-range communication technologies often combined with WBAN systems. As a consequence, this research work presented a review on the newly emerged LPWAN communication systems, which include the proprietary and non-proprietary LPWAN based communication systems, which were reviewed to determine their suitability for WBANs. Recommendations that are suitable for improving the efficiency of the next-generation WBAN systems are given. It is noteworthy to mention that, based on the unique features of the LPWAN solutions discussed in this study, such as low energy consumption, low latency, wide-area communication coverage, and health data transmission reliability, the LPWAN solutions can be considered suitable candidates for remote HCM in WBAN systems, to achieve efficient data communications. However, to guarantee the fruitfulness of the LPWAN solutions in WBAN systems, a number of critical issues, including the development of energy-efficient communication protocols, as well as improving data latency and also data-transmission-rate needs to be addressed. The aforementioned issues are discussed as open research problems.

## Figures and Tables

**Table 1:** Comparison of related survey works on low-power wide-area networks (LPWAN) communication networks in health-care monitoring (HCM) and implementation design.

Reference	Focus on Short-Range Technologies	Concept of LPWAN Solution in HCM	Concept of LPWAN Solution in WBANs	LPWAN Implementation Design WBANs
[5]	✓	X	X	X
[6]	✓	X	X	X
[8]	✓	X	X	X
[10]	✓	X	X	X
[13]	✓	X	X	X
[14]	✓	X	X	X
[15]	✓	X	X	X
[16]	X	✓	✓	X
[17]	X	✓	✓	X
[18]	✓	X	X	X
[19]	✓	X	X	X

**Table 2:** Comparison of LPWAN communication systems.

Communication Solution Parameters	Proprietary-Based LPWAN Communication Systems			Non-Proprietary Based LPWAN Communication Systems		
	RPMA/Ingenu	Sigfox	LoRa	LTE-M1	EC-GSM	NB-IoT
Transmission power	__	14 dBm [54]	15 dBm [54]	20 dBm [33]	33 dBm [33]	20 dBm or 23 dBm [54]
Battery lifespan	10 years	5 years [49]	10 years [49]	10 years [49]	10 years [49]	More than 10 years [45,56]
Data rate	20 kbps [57]	100 bps [48]	50 kbps [57]	1 Mbps [48]	10 kbps	Downlink: 160–250 kbps Uplink: 160–200 kbps [45]
Latency of health data	10 s [58]	10 s [58]	10 s [58]	150 ms [58]	__	<10 s [58]
Communication range/Link budget	Rural: 10 km Urban: 3 km/168 dB	Rural: 50 km Urban: 10 km/160 dB	Rural: 15 km Urban: 5 km/157 dB [49]	Rural: 15 km Urban: -/155.7 dB	Rural: 15 km Urban:-/164 dB (33 dBm) and 154 dB(23 dBm)	Rural: 35 km Urban: 8 km/164 dB [48]
Topology/Network	Star, tree/WAN	Star/WAN	Star/WAN	Star/WAN	Star/WAN	Star/WAN
Deployment cost	High	High	High	Low	Low	Low
Carrier frequency	2.4 GHz ISM free-licensed band	Free-licensed Sub-GHz ISM band	Free-licensed Sub-GHz ISM band	Licensed Sub-GHz	Licensed Sub-GHz	Licensed Sub-GHz
Network capacity per cell	More than 50,000 sensor devices	50,000 sensor devices	40,000 sensor devices	20,000 sensor devices	50,000 sensor devices	More than 50,000 sensor devices
Security mechanism	AES 256-bit, 16 B hash [16]	140 message limit per day, scrambling techniques and encryption, signing message with private key	AES encryption	Authentication method using MME [35]	Supports 3GP Psecurity mechanism [57]	Supports the 3GPP S3 mechanisms, such as device identification, identity confidentiality, health data integrity, and authentication
Modulation scheme	CDMA, RPMA-DSSS	DBPSK or GFSK or UNB	CSS	Downlink: FDMA Uplink: C-FDMA	Downlink and Uplink TDMA or FDMA, GMSK and 8PSK	Downlink: OFDMA Uplink: SC-FDMA
Mobility support	Limited support [56]	No support [56]	Support [56]	Support [56]	Support [56]	No support [56]
Advantage	Support long communication range, energy efficiency, and low operational cost	Support long communication range, energy efficiency, and low operational cost	Support long communication range, energy efficiency, and low operational cost	Support remote health-care monitoring, energy efficient, provide fast and reliable network	Support remote health-care monitoring, energy efficient,	Support remote health-care monitoring, energy efficient provide fast and reliable network
Disadvantage	Interference issue, high deployment cost, and low health data reliability	Interference issue, high deployment cost, and low health data reliability	Interference issue, high deployment cost, and limited network size	Limited network capacity	Extremely low data rate	High maintenance and operational cost since it is on the licensed spectrum, also cost of SIM card purchase.
WBANs suitability	Low	Low	Moderate	High	Low	High

**Table 3:** QoS requirements for medical and nonmedical applications in WBANs.

Application Type	Sensor Devices	Data Rate	Frequency (Hz)	Accuracy (bits)	Energy Consumption	Latency (ms)	Reference
Implantable medical application	Peacemaker	<1 kbps	<500 Hz	12	Low	<150	[70,71,72]
	Endoscope capsule	>2 Mbps	__	__	High	<150	
	Glucose level sensor	<1 kbps	< 50 Hz	16	Very low	<150	
	Drug delivery capsule	<320 kbps	__	__	Low	<150	
	Brain depth simulator	<16 kbps	130 Hz	__	__	<150	
	Cochlear implant	<1Mbps	5, 12, 49 MHz	__	__	<150	
Wearable medical application	Blood pressure	<10 kbps	<100 Hz	12	High	<150	[73,74]
	ECG	3 kbps	<500 Hz	12	High	<150	
	Blood flow rate	480 kbps	<40 Hz	12	Low	<150	
	Temperature	120 bps	<1Hz	12	Low	<150	
Nonmedical application	Audio streaming	1 Mbps	__	__	High	<250	[75]
	Video streaming	<10 Mbps	__	__	High	<250	
	Voice	100 kbps	__	__	__	<250	
	Motion sensor	4.8–35 kbps	30–100 Hz (depending on the activity recognition or other tasks)	12–16	__	<250	
